# Supplementary material for: Development and Validation of a Prognostic Model for Lung Adenocarcinoma Based on CAF-Related Genes: Unveiling the Role of COX6A1 in Cancer Progression and CAF Infiltration
Source: Int J Mol Sci. 2025 Apr 8;26(8):3478. doi: 10.3390/ijms26083478 (PMC12026577; doi:10.3390/ijms26083478)
Supplement: Supplementary file 1 [file ijms-26-03478-s001.zip › ijms-3507345-supplementary.pdf]

Supplementary Materials

Table S1. Demographic and clinical characteristics of GSE31210 dataset.

Characteristics		GSE31210
Stage	Stage I	168 (74.34%)
	Stage II/III	58 (25.66%)
MYC_copy	≤1	57 (25.22%)
	>1	169 (74.78%)
Smoke_history	Never-smoker	115 (50.88%)
	Ever-smoker	111 (49.12%)
Age	≤60	108 (47.79%)
	>60	118 (52.21%)
Gender	Male	105 (46.46%)
	Female	121 (53.54%)
time	≤5	124 (54.87%)
	>5	102 (45.13%)
status	0	191 (84.51%)
	1	35 (15.49%)

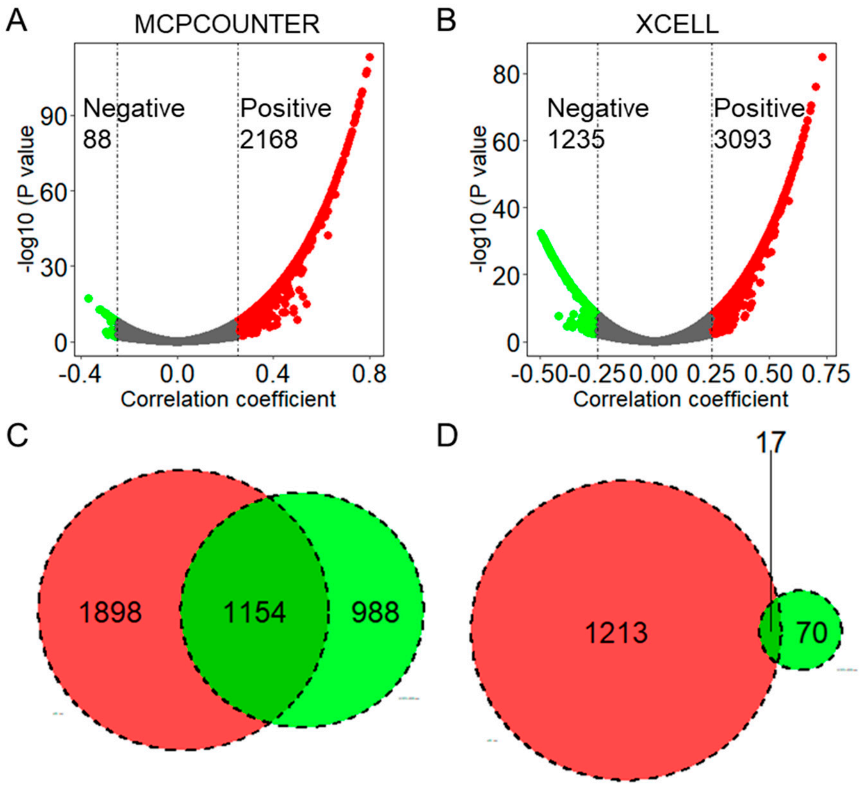

Figure S1. Identification of CAF Infiltration-Related Genes. Scatter plots show the correlation between CAF infiltration scores and the expression of all genes in the TCGA LUAD dataset, calculated using the MCPCOUNTER (A) and XCELL (B) algorithms. The Venn diagrams illustrate the overlap of genes that are positively (C) and negatively (D) correlated with CAF infiltration scores, based on the two algorithms. TCGA: The Cancer Genome Atlas; LUAD: Lung adenocarcinoma; CAF: Tumor-associated fibroblast.

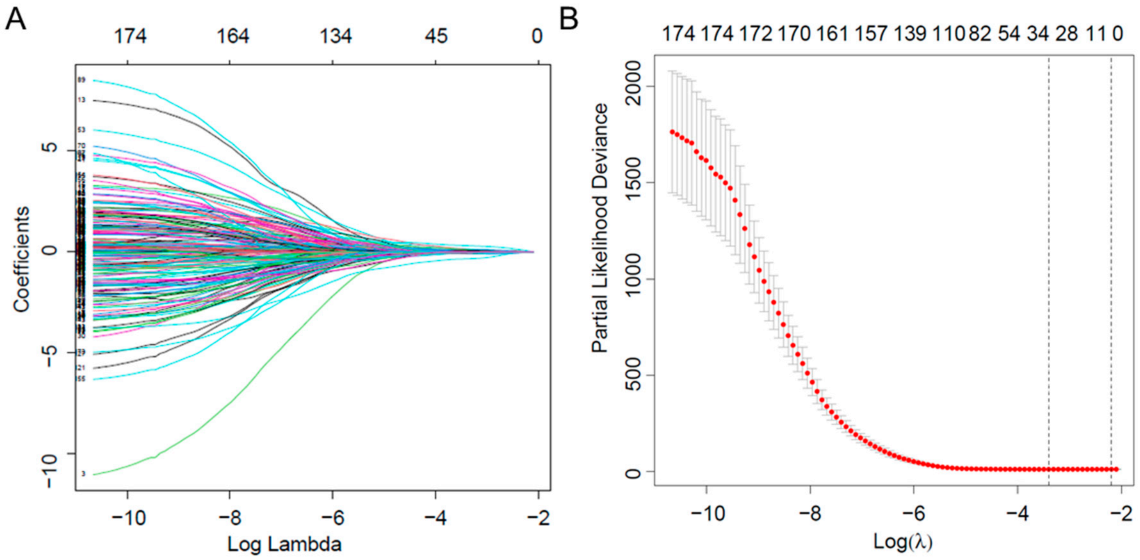

Figure S2. (A,B) LASSO Cox regression analysis identified a total of 30 CAFRGs for overall survival.

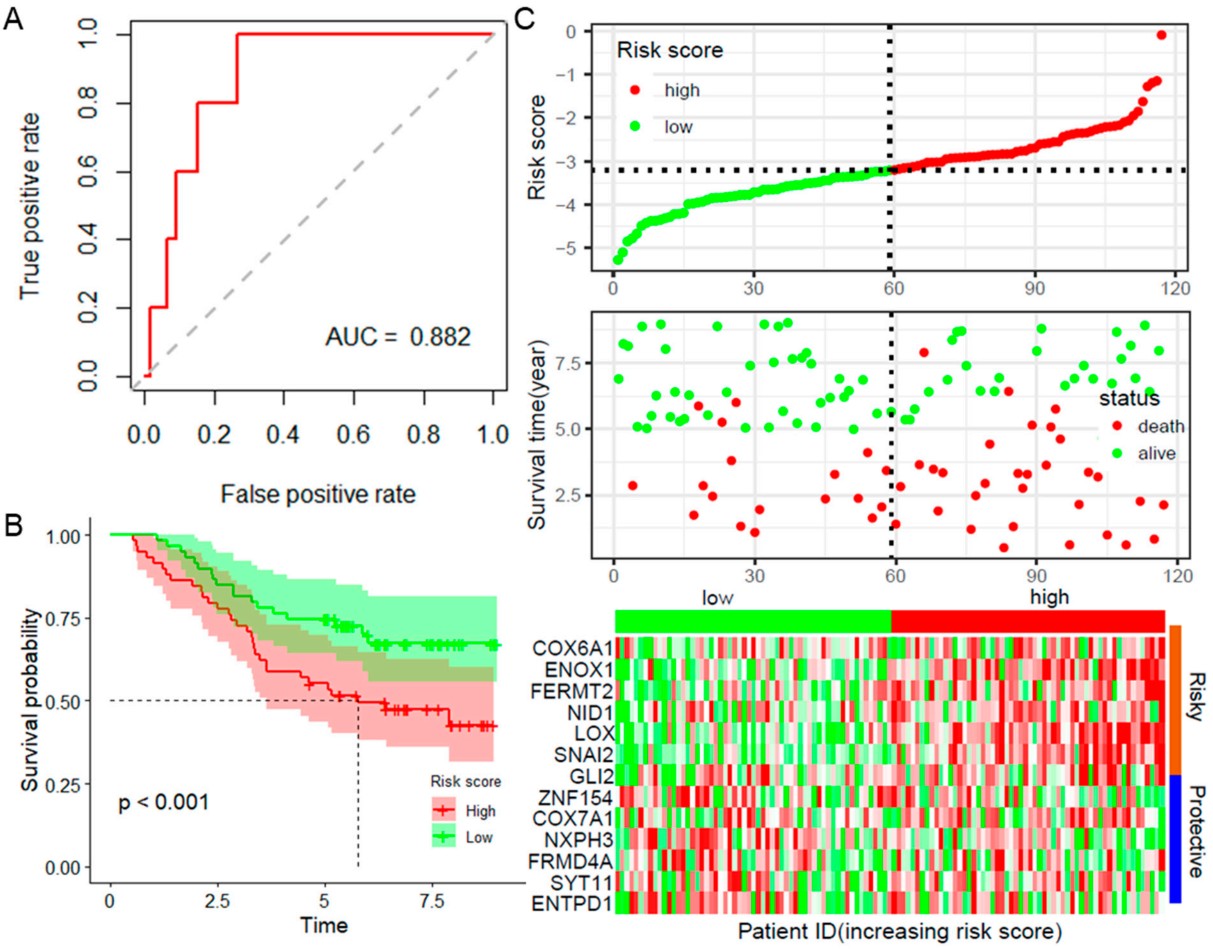

Figure S3. Validation of the Model's Robustness in GSE13213. (A) ROC curve showing ROC and AUC values for 1 year in the GSE13213 dataset; (B) Survival curves comparing high-risk and low-risk patients in the GSE13213 dataset; (C) Distribution of risk scores and survival outcomes in high- and low-risk samples in the GSE13213 dataset. ROC: Receiver Operating Characteristic; AUC: Area Under the Curve.

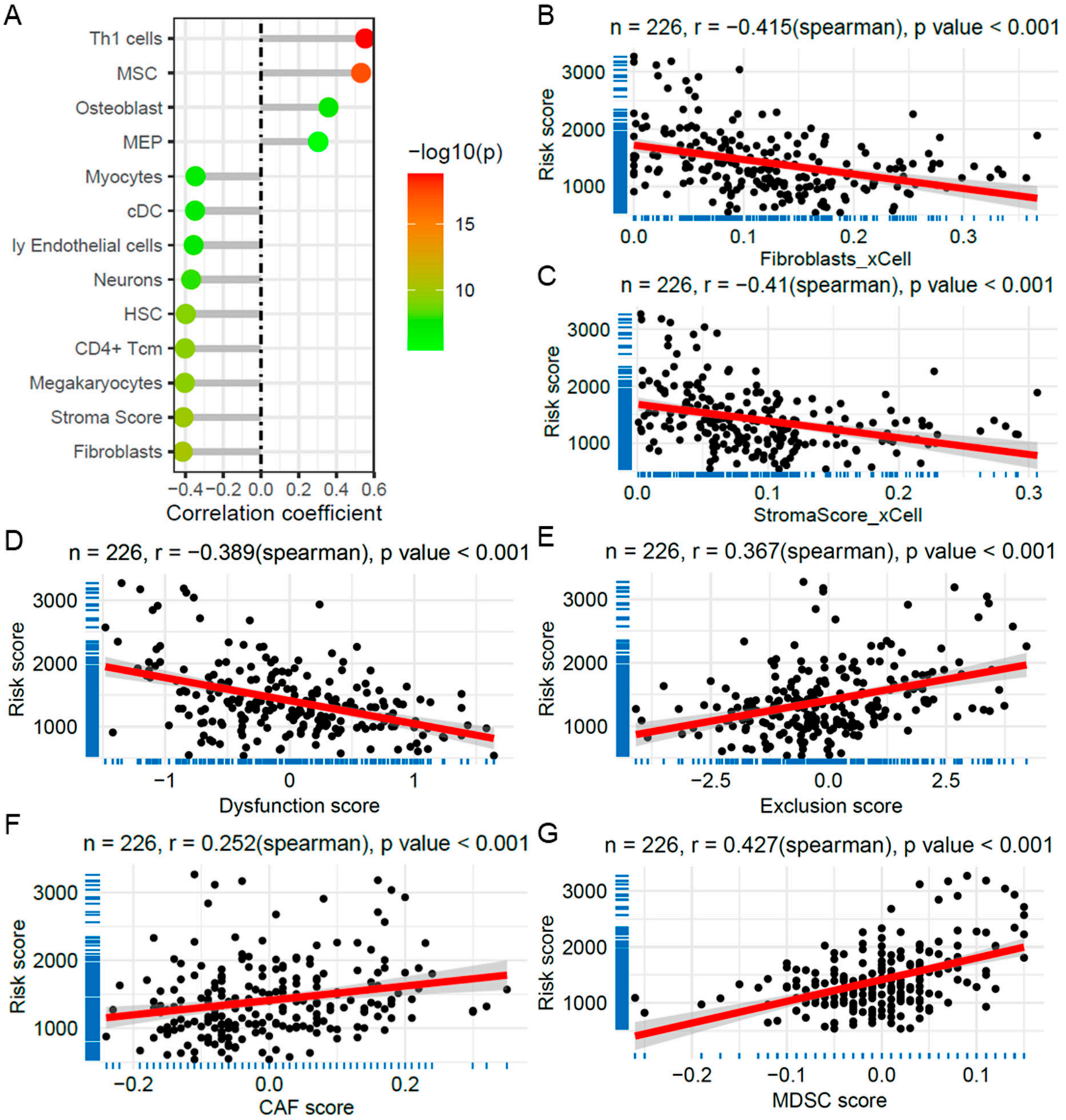

Figure S4. Correlation of Risk Score with Immune Cell Infiltration and Immunotherapy in GSE31210 Dataset. (A) Lollipop plot showing the correlation between risk score and immune cell infiltration scores calculated by the xCell algorithm; Scatter plots depicting the correlation between risk score and fibroblasts infiltration score (B) and stroma score (C). Scatter plots demonstrating the correlation between risk score and immune infiltration scores of T cell dysfunction (D), exclusion (E), CAF (F), and MDSC (G) based on the TIDE algorithm. TIDE: Tumor Immune Dysfunction and Exclusion; CAF: Cancer-Associated Fibroblasts; MDSC: Myeloid-Derived Suppressor Cells.

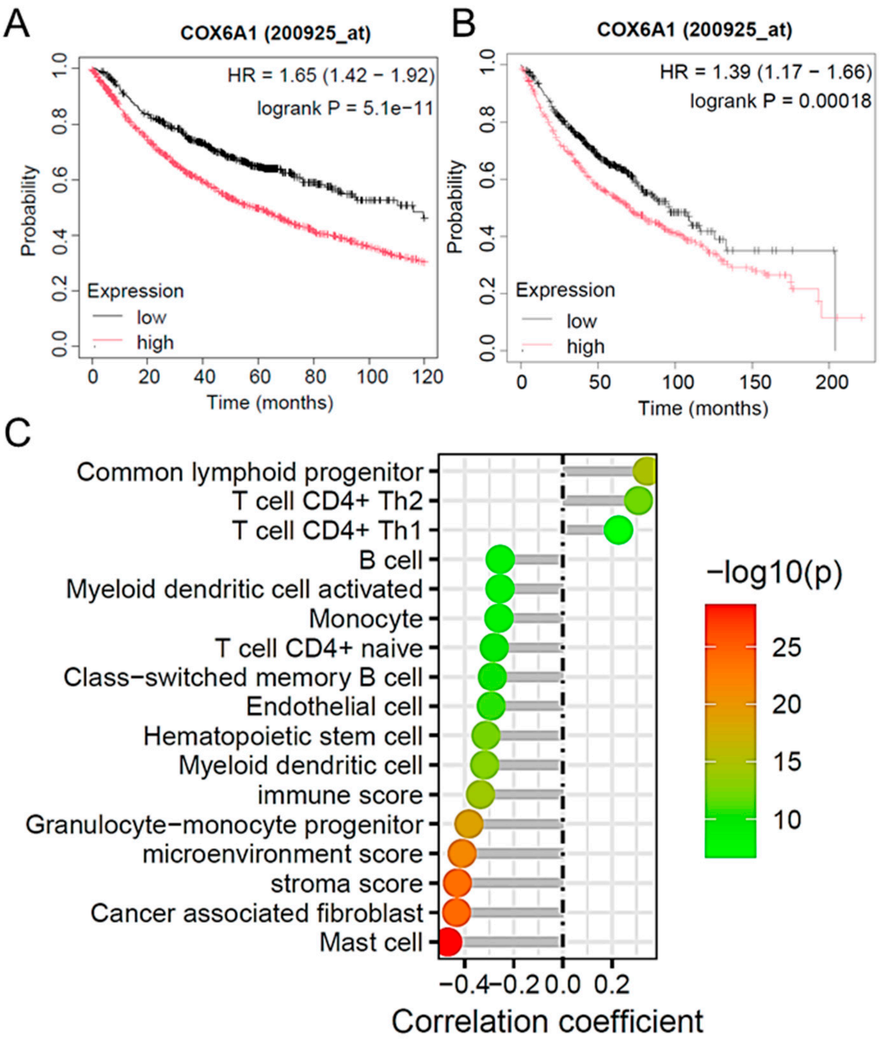

Figure S5. COX6A1 contributes to poor prognosis and relates to immune cells infiltration. (A) The KM survival curve of overall survival (A) and first progression (B) for patients with high and low expression of COX6A1 based on KM plotter tool. (C) The lollipop plot illustrates the correlation between COX6A1 expression and immune cell infiltration scores derived from xCell.
